# The cell wall and the response and tolerance to stresses of biotechnological relevance in yeasts

**DOI:** 10.3389/fmicb.2022.953479

**Published:** 2022-07-28

**Authors:** Ricardo A. Ribeiro, Nuno Bourbon-Melo, Isabel Sá-Correia

**Affiliations:** ^1^Institute for Bioengineering and Biosciences, Instituto Superior Técnico, Universidade de Lisboa, Lisbon, Portugal; ^2^Department of Bioengineering, Instituto Superior Técnico, Universidade de Lisboa, Lisbon, Portugal; ^3^Associate Laboratory i4HB—Institute for Health and Bioeconomy at Instituto Superior Técnico, Universidade de Lisboa, Lisbon, Portugal

**Keywords:** cell wall, yeasts of biotechnological relevance, stress response, stress tolerance, signaling pathways

## Abstract

In industrial settings and processes, yeasts may face multiple adverse environmental conditions. These include exposure to non-optimal temperatures or pH, osmotic stress, and deleterious concentrations of diverse inhibitory compounds. These toxic chemicals may result from the desired accumulation of added-value bio-products, yeast metabolism, or be present or derive from the pre-treatment of feedstocks, as in lignocellulosic biomass hydrolysates. Adaptation and tolerance to industrially relevant stress factors involve highly complex and coordinated molecular mechanisms occurring in the yeast cell with repercussions on the performance and economy of bioprocesses, or on the microbiological stability and conservation of foods, beverages, and other goods. To sense, survive, and adapt to different stresses, yeasts rely on a network of signaling pathways to modulate the global transcriptional response and elicit coordinated changes in the cell. These pathways cooperate and tightly regulate the composition, organization and biophysical properties of the cell wall. The intricacy of the underlying regulatory networks reflects the major role of the cell wall as the first line of defense against a wide range of environmental stresses. However, the involvement of cell wall in the adaptation and tolerance of yeasts to multiple stresses of biotechnological relevance has not received the deserved attention. This article provides an overview of the molecular mechanisms involved in fine-tuning cell wall physicochemical properties during the stress response of *Saccharomyces cerevisiae* and their implication in stress tolerance. The available information for non-conventional yeast species is also included. These non-*Saccharomyces* species have recently been on the focus of very active research to better explore or control their biotechnological potential envisaging the transition to a sustainable circular bioeconomy.

## Introduction

*Saccharomyces cerevisiae* is an essential eukaryotic cell model that also has a plethora of industrial applications (Parapouli et al., 2020). Since early in human civilization, it has been extensively used in the production of fermented food and beverages, which nowadays include bread, chocolate, wine, beer, cider, sake, spirits (rum, vodka, whisky, brandy), and other alcoholic beverages arising from the fermentation of fruits, honey, and tea (Walker and Stewart, 2016; Parapouli et al., 2020). Nowadays, many enzymes, pharmaceuticals, nutraceuticals, and other added-value bioproducts can be produced in engineered yeast cell factories (Borodina and Nielsen, 2014; Runguphan and Keasling, 2014; Kwak and Jin, 2017; Nielsen, 2019; Fathi et al., 2021; Wang et al., 2021; Arnesen et al., 2022; Stovicek et al., 2022). The implementation of a sustainable circular bioeconomy requires the development of Advanced Yeast Biorefineries to produce biofuels (e.g., bioethanol and biodiesel), chemicals, materials, and other bioproducts from organic residues from agriculture, forestry, and industry residues (Madhavan et al., 2017; Mukherjee et al., 2017; Yamakawa et al., 2018; Panahi et al., 2019; Saini and Sharma, 2021; Usmani et al., 2021; Raj et al., 2022). For the transition to a sustainable biobased economy, the use of non-conventional yeasts is gaining momentum since strains of this heterogenous group of non-*Saccharomyces* species are advantageous alternatives to *S. cerevisiae* whenever they can natively express highly interesting metabolic pathways, assimilate a wider range of carbon sources, and/or exhibit higher tolerance to relevant bioprocess-related stresses (Thorwall et al., 2020). Non-conventional yeasts are also being explored to enhance the flavor profiles and reduce the ethanol content of alcoholic beverages (Gschaedler, 2017; Holt et al., 2018; Bourbon-Melo et al., 2021). Spoilage yeasts, as is the case of the osmophilic and highly weak acid tolerant yeast species of the *Zygosaccharomyces* genera, are also being studied due to their high tolerance to stresses associated to food preservation (Palma and Sá-Correia, 2019). However, the advanced genome-editing techniques and other genomic and bioinformatics information and tools available for *S. cerevisiae*, are arguably the major reason why this species is still considered the major yeast cell factory (Nandy and Srivastava, 2018; Nielsen, 2019).

Regardless of the yeast species or the specific application, industrial yeasts typically encounter stresses that trigger intricate cellular responses involving multiple players (Bauer and Pretorius, 2000; Gibson et al., 2007; Eardley and Timson, 2020). Among these players is the cell wall. The cell wall maintains cell shape and integrity and, together with the plasma membrane, is part of the cell envelope and the first line of defense against multiple adverse environmental conditions (Stewart, 2017). The cell wall is a highly dynamic organelle whose biochemical and biophysical properties can be finely tuned as yeast cells encounter different stresses throughout the course of industrial bioprocesses (Klis, 2002; Stewart, 2017). Considerable cell energy must be invested for maintaining such complex and energetically expensive response, considering that over 1200 genes are estimated to be involved, directly or indirectly, in cell wall synthesis and regulation (De Groot et al., 2001). A deeper mechanistic insight into how the cell enacts such changes at the level of the cell wall is expected to lead to a better understanding of yeast’s adaptation to stresses of industrial relevance that ultimately may lead to improved bioprocess productivity and product yield. This understanding is also instrumental to guide the improvement of food preservation practices, in particular those involving the use of weak acid food preservatives, temperature- and osmotic- induced-stresses for the microbiological stabilization and conservation of foods, beverages, and other goods.

The suggested role for the cell wall in yeast stress response and tolerance, mostly emerged from the datasets obtained from the numerous genome-wide expression analyses or chemogenomic analyses under stress reported in recent years. However, the cell wall has not received the attention it deserves as a tolerance determinant toward multiple stresses. The objective of this review paper is to gather the literature available on the topic and provide a critical opinion and a comprehensive view on the current knowledge on the role of the cell wall and related signaling pathways in yeast adaptation and survival under industrially relevant stresses. Attempts of stress tolerance improvement through the manipulation of cell wall biosynthetic pathway are also included. Most of the published knowledge pertains to the yeast model *S. cerevisiae* but the information available in the scientific literature for other yeast species of biotechnological relevance is also offered.

## The yeast cell wall: composition, structure, function, and biosynthesis

**FIGURE 1:**
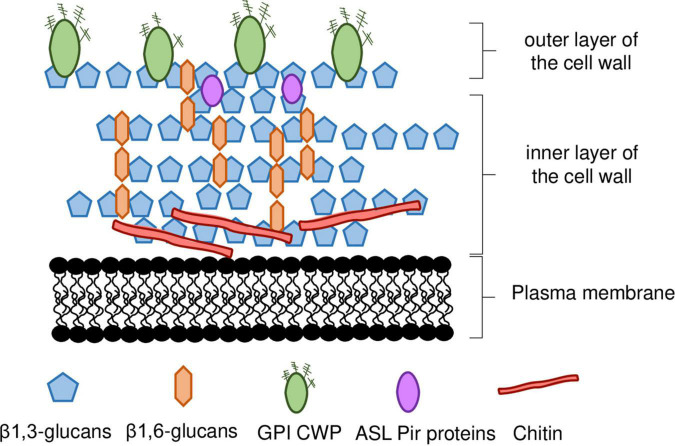
Schematic representation of *S. cerevisiae* cell wall. The inner layer of the cell wall is mostly composed of β1,3-glucan chains branched with β1,6-glucans, and chitin. The outer layer is composed of mannoproteins, most of which are linked to the inner wall by a GPI anchor, whereas ASL-Pir proteins seem to be uniformly distributed throughout the inner layer. ASL-CWP, alkali-sensitive linkage cell wall protein. GPI-CWP, glycosylphosphatidylinositol cell wall protein. Details in the main text.

The cell wall of *S. cerevisiae* represents up to 30% of the cell’s dry weight (w/w) being almost entirely composed of polysaccharides (≈85%) and proteins (≈15%) (Nguyen et al., 1998) with a thickness of 115—120 nm, as determined by atomic force microscopy and ultrathin-sectioning electron microscopy (Dupres et al., 2010; Yamaguchi et al., 2011). It is a layered structure with two different layers (Figure 1), distinguishable by ultrathin-sectioning electron microscopy (Hagen et al., 2004; Yamaguchi et al., 2011; Orlean, 2012). The electron-dense outer layer mostly consists of mannoproteins whereas, in the more electron-transparent inner layer, glucans are the major component and chitin is in a lesser extent (Hagen et al., 2004; Yamaguchi et al., 2011; Orlean, 2012). Most of the cell wall mechanical strength derives from its inner layer, in which the β-linked glucans are the major components of the polysaccharide fraction, and alone represent 30–60% of the cell wall dry weight (Fleet, 1991). Approximately 30–60% of the cell wall dry biomass is composed by β-glucans, around 85% of these are 1,3-β-glucans and the remaining are 1,6-β-glucans (Aguilar-Uscanga and François, 2003; Orlean, 2012; Figure 1). The β1,3-glucan chains are composed of ≈1500 glucose units and are assembled in coiled spring-like structure that confer elasticity and tensile strength to the cell wall (Klis, 2002). The β1,6-glucans chains are shorter than the β1,3-glucan chains and, although quantitatively being a minor component of the wall, β1,6-glucans have a central role in cross-linking β1,3-glucans together (Kollár et al., 1997; Orlean, 2012). Additionally, β1,6-glucans can be connected to mannoproteins with a glycosylphosphatidylinositol (GPI) anchor and to chitin (Kollár et al., 1995; Orlean, 2012). The reducing ends of β1,3-glucans chains can be linked to a side-branching β1,6-glucan on β1,3-glucans chains, forming a fibrillar structure that serves as backbone and anchorage point for other constituents of the cell wall (Lesage and Bussey, 2006; Figure 1). The non-reducing ends of 1,3-β-glucans are linked to the reducing ends of chitin through a β-1,4 link (Kollár et al., 1995, 1997; Orlean, 2012).

Chitin is a linear polymer of β1,4-linked *N*-acetylglucosamine (GlcNAc), representing less than 2% of *S. cerevisiae* cell wall dry weight (Figure 1). Chitin can occur both in the free form, or bound to β-glucans (Kollár et al., 1995, 1997; Orlean, 2012). Chitin is normally concentrated as a ring between the mother cell and the emerging bud and in the lateral walls of the mother cell after septation (Cabib, 2009). In response to stress, chitin levels can increase to as much as 20% of the cell wall dry weight (Popolo et al., 1997; Dallies et al., 1998; Ram et al., 1998; Osmond et al., 1999; Valdivieso et al., 2000; Magnelli et al., 2002; Orlean, 2012). Changes occurring in cell wall nanomechanical properties, such as cell surface stiffness, are mostly dependent on the crosslinking between β-glucans and chitin (Dague et al., 2010) and occur in response to stresses of industrial relevance (Dague et al., 2010; Pillet et al., 2014; Schiavone et al., 2016; Ribeiro et al., 2021, 2022).

The outer layer is composed by cell wall mannoproteins (Figure 1). These mannoproteins are heavily glycosylated, modified with both N-and O-linked carbohydrates, commonly formed by mannose (Klis et al., 2006; Schiavone et al., 2014). The outer layer serves an important protective role in the cell by limiting the access of external aggressors, such as foreign enzymes, to the inner layer (Klis et al., 2006). The proteins that constitute this layer are involved in a wide range of functions often related to cell-to-cell interactions (e.g., mating, flocculation, biofilm formation, etc.) (Klis, 2002). Increased cell wall hydrophobicity influences flocculation leading to increased robustness to inhibitory chemical compounds (Kahar et al., 2022). This was associated with changes in the expression of *MOT3* gene, encoding a transcription regulator that controls the expression of a cell wall protein (CWPs) encoding gene, *YGP1*, that influences cell wall hydrophobicity (Kahar et al., 2022).

There are two major classes of CWPs, namely the glycosylphosphatidylinositol CWPs (GPI-CWPs), and the alkali-sensitive linkage CWPs (ASL-CWPs), which include proteins of the internal repeats (Pir) family (Klis, 2002; Klis et al., 2006; Figure 1). While GPI-CWPs are typically linked to β1,6-chains by a GPI anchor, ASL-CWPs are directly linked to β1,3-glucans through an alkali-labile bond (Lesage and Bussey, 2006). They also differ in their distribution throughout the cell wall. While GPI-CWPs are found in the outer layer, Pir-CWPs seem to be uniformly distributed throughout the inner layer, which is consistent with their direct association with β1,3-glucans (Kapteyn et al., 1999b; Klis et al., 2006; Figure 1).

**FIGURE 2:**
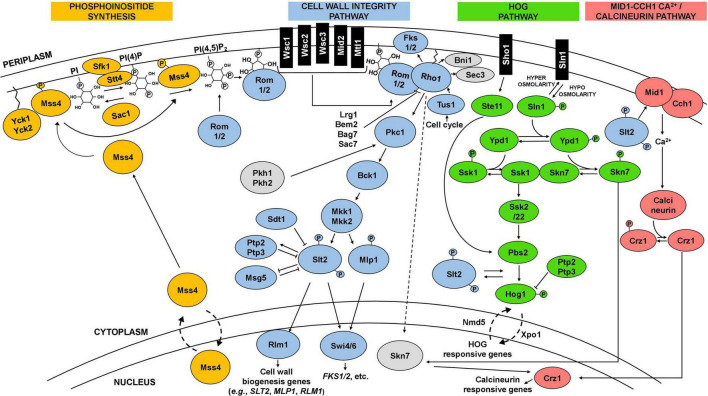
Schematic representation of the CWI, HOG, and calcineurin signaling pathways and interactions. The cell wall integrity pathway (CWI), in blue, is the main pathway involved in the maintenance of yeast cell wall integrity. It is frequently activated by a signal starting at plasma membrane sensors (Wsc1-3, Mid2, Mtl1), which then trigger a cascade culminating in the phosphorylation of Slt2. Phosphorylated Slt2 activates the transcription factor Rlm1 and the SBF complex (Swi4/6), triggering a transcriptional response that elicit changes at cell wall level. Phosphoinositide synthesis (in orange) plays an important role in the activation of the CWI pathway by recruiting Rom1/2 to the plasma membrane, which activates the main regulator of the pathway, the GTPase Rho1. The CWI pathway receives input and interplays with other pathways, including the high osmolarity glycerol (HOG) pathway (in green) and the calcineurin pathway (in pink), represented in the figure, but also with the protein kinase A (PKA) and TOR signaling pathways. Details in the text.

Cell wall biogenesis involves cell wall polysaccharide synthases and enzymes involved in cell wall remodeling, assembly and degradation (Klis et al., 2006; Free, 2013; Figure 2). The β1,3-glucans are synthetized as a linear polymer by the *FKS* family of genes (*FKS1-3)*. The *FKS1* and *FKS2* genes code for β1,3-glucan synthases and differ mostly on the expression pattern, whereas *FKS3* remains poorly characterized (Garrett-Engele et al., 1995; Mazur et al., 1995; Igual et al., 1996; Zhao et al., 1998; Lesage and Bussey, 2006). While *FKS1* expression is prevalent under optimal growth, *FKS2* expression is induced in response to different stresses such as glucose depletion, alternative carbon sources (e.g., acetate, glycerol, galactose), high extracellular Ca^2+^ or heat stress (Garrett-Engele et al., 1995; Mazur et al., 1995; Igual et al., 1996; Zhao et al., 1998). Several genes influence β-1,6-glucan levels, in particular the *KRE6* and *SKN1* genes, encoding proteins involved in β1,6-glucan synthesis, and the *KRE9* and *KHN1* encoding CWPs presumably involved in β1,6-glucan cross-link to other components of the cell wall (Roemer et al., 1994; Lesage and Bussey, 2006). Chitin is synthetized as a linear polymer by the chitin synthases encoded by the *CHS1-3* gene family. The gene *CHS3*, encoding chitin synthase III, is by far responsible for the majority of chitin synthesis, whether it is during optimal growth conditions or during stress response when increased chitin deposition in the cell wall occurs (Roncero, 2002; Klis et al., 2006; Lesage and Bussey, 2006; Orlean, 2012). Also, CWPs mannosylation requires several genes encoding proteins with mannosyltransferase activity (Gonzalez et al., 2009a; Orlean, 2012). The *GAS* family (*GAS1-*5), encodes β1,3-glucanosyltransferases involved in β1,3-glucan branching and elongation, in which *GAS1* has a major role and is also required for β1,6-glucan linkage with β1,3-glucan chains (Ram et al., 1998; Ragni et al., 2007; Orlean, 2012; Aimanianda et al., 2017). Shortening of glucan chains is also required for cell wall remodeling and involves *BGL2* and *EXG1-2* encoding a major endoglucanase and major exoglucanases, respectively (Larriba et al., 1995; Lesage and Bussey, 2006; Orlean, 2012; Aimanianda et al., 2017). The transglycosylases GPI proteins encoded by the *CRH1-2* genes have a major role in the cross-linkage between chitin and glucans (Cabib et al., 2007, 2008; Cabib, 2009; Orlean, 2012; Blanco et al., 2015). Endochitinases, encoded by *CTS1-2*, are required for septation and cell separation (Kuranda and Robbins, 1991; Cabib et al., 1992; Orlean, 2012).

*Saccharomyces cerevisiae* cell wall appears to have many organizational similarities with the walls of other ascomycetous yeasts and even of basidiomycetous yeasts (Jaafar and Zueco, 2004; De Groot et al., 2005, 2008; Patel and Free, 2019; Garcia-Rubio et al., 2020). However, there are differences between the cell walls of *S. cerevisiae* and other fungi. For instance, the cell wall of *S. cerevisiae* does not contain α-glucans, chitosan, or melanin, and its chitin content is relatively low, especially when compared to other yeasts or filamentous fungi (e.g., *Candida albicans, Aspergillus niger, Paracoccidioides brasiliensis, Blastomyces dermatitidis*) (Kanetsuna et al., 1969; Garcia-Rubio et al., 2020; van Leeuwe et al., 2020).

## Cell wall-related signaling pathways induced by stress

Yeast cells sense and respond to environmental stresses through the induction and activity of different signaling pathways (Chen and Thorner, 2007). Depending on the type of stress, specific pathways can be triggered and directly, or indirectly, elicit changes in the composition and architecture of the cell wall (Levin, 2011; Figure 2). The CWI signaling pathway is one of the most well-described pathways, characterized for its role in cell wall maintenance and homeostasis, particularly in response to environmental external stimuli that may damage the cell wall (Gustin et al., 1998; Levin, 2011; Sanz et al., 2018; Figure 2). CWI pathway relies on a family of plasma membrane sensors, namely Wsc1-3, Mid2, and Mtl1. Stimuli that may impose a stress to the cell are detected by these sensors and triggers a phosphorylation cascade that elicit transcriptional changes to enable the cell to adapt to stress conditions (Kock et al., 2016; Sanz et al., 2018; Figure 2). For the Wsc1 sensor, the signaling transduction to the downstream components of the pathway is influenced by the spatial distribution of these sensors within the plasma membrane, requiring a functional extracellular cysteine-rich domain to form clusters in specific microdomains or rafts (Heinisch et al., 2010; Schöppner et al., 2022). Industrially relevant stresses reported to trigger a response by the referred CWI pathway membrane sensors are highlighted in Table 1. Together with phosphatidylinositol 4,5-biphosphate, PIP_2_, which recruits the guanine nucleotide exchange factors (GEFs) Rom1 and Rom2, the surface sensors activate the Rho1 GTPase (Levin, 2011; Sanz et al., 2018). Rho1 regulates both β1,3-glucan synthases encoded by *FKS1* and *FKS2*, and β1,6-glucan synthase activities, and is considered a key regulator of CWI signaling (Levin, 2011). Rho1 activates Pkc1 and sets in motion a series of phosphorylation events which sequentially activate Bck1, Mkk1/2, and the pathway’s MAPK, Slt2 (Levin, 2011; Figure 2). Finally, Slt2 activates two transcription factors, Rlm1 and SBF (Swi4/Swi6 complex), that coordinate the CWI transcriptional response (Levin, 2011; Orlean, 2012; Figure 2). This response mostly involves the activation of cell wall biogenesis genes. The Rlm1 transcription factor is responsible for the expression of most of the genes induced in response to cell wall stress, such as the *CHS3* gene, and also regulates the expression of *FKS1* (Jung and Levin, 1999; Klis et al., 2006). Importantly, Rlm1 activates *MLP1*, coding for a Slt2 pseudo-kinase paralog, that, together with Slt2, activates the SBF complex for transcription of a subset of cell wall stress-activated genes. Among them are genes related with glucan synthesis (*FKS1, FKS2)* or glucan elongation and branching (*GAS1*) (Igual et al., 1996; Baetz et al., 2001; Kim et al., 2008; Levin, 2011; Harris et al., 2013)This brief description of the CWI pathway as a simple linear cascade of events can be deceiving. In fact, the CWI pathway receives lateral influences from cAMP-Protein Kinase A (PKA) signaling, TOR signaling, calcineurin signaling, the HOG pathway, and likely from others not yet clarified (Fuchs and Mylonakis, 2009; Rodríguez-Peña et al., 2010; García et al., 2017; Udom et al., 2019; Jiménez-Gutiérrez et al., 2020a,b; Figure 2). This complex network is what enables the CWI pathway to be activated by numerous types of stressors, ensuring an adequate response to each stress or group of stresses (Figure 2). The existence of a complex interplay between the CWI pathway and other signaling pathways is also being revealed for other yeast species (Donlin et al., 2014; Valiante et al., 2015; Madrid et al., 2016; Assis et al., 2018).

**TABLE 1:** Cell wall integrity membrane sensors implicated in the sensing of industrially relevant stresses.

CWI membrane sensor	Industrially relevant stress	Bibliographic references
Wsc1	Hyper-osmolorarity Heat Acetic acid Alkaline pH Diamide and H_2_O_2_	García-Rodríguez et al., 2005 Rajavel et al., 1999 Mollapour et al., 2009 Serrano et al., 2006; Kwon et al., 2016 Vilella et al., 2005
Wsc2	H_2_O_2_	Vélez-Segarra et al., 2020
Wsc1/Wsc2	Impaired mannosylinositol phosphorylceramide metabolism	Tanaka and Tani, 2018
Mid2	Hyper-osmolorarity Heat Low pH – media acidified with a strong acid H_2_O_2_	García-Rodríguez et al., 2005 Rajavel et al., 1999 Claret et al., 2005 Jin et al., 2013
Mtl1	Diamide and H_2_O_2_	Vilella et al., 2005; Jin et al., 2013

Changes occurring at the level of plasma membrane can also elicit responses at the level of the cell wall. Plasma membrane stretching appears to be the main factor in activating the CWI pathway in response to a number of stresses, including high osmotic pressure and supra-optimal temperature (Kamada et al., 1995; Hohmann, 2002b). This is consistent with the CWI pathway-activating sensors being located at the membrane and the likely role of Wsc1 as a mechanosensor (Kock et al., 2015). Alterations of plasma membrane lipid composition have also been shown to impact the CWI pathway, namely, a defective biosynthesis of the complex sphingolipid mannosylinositol phosphorylceramide (MIPC) leads to increased abundance of the phosphorylated form of Slt2 and sensitivity to cell wall-perturbing agents (Morimoto and Tani, 2015). This phenotype is partly suppressed by the upregulation of ergosterol biosynthesis, suggesting that MIPC and ergosterol are coordinately involved in maintaining the integrity of the cell wall (Tanaka and Tani, 2018). Also, a reported crosstalk between plasma membrane ergosterol content (related with the level of expression of the plasma membrane ergosterol transporter Pdr18), and cell wall biophysical properties suggested a coordinated response to counteract acetic acid deleterious effects, reinforcing the notion that plasma membrane lipid composition influences cell wall integrity during stress (Ribeiro et al., 2022).

For the main and better studied stresses encountered during industrial bioprocesses, this review provides, whenever possible, an integrative view of the pathways and responses that cooperatively maintain cell wall integrity and, in so doing, helps yeasts to resist to multiple stresses.

## Yeast response and tolerance to industrially relevant stresses involving the cell wall

### The cell wall in the response to oxidative stress

All aerobically growing organisms suffer exposure to oxidative stress, caused by reactive oxygen species (ROS) capable of damaging cellular DNA, lipids, carbohydrates, and proteins, threatening cell integrity (Jiménez-Gutiérrez et al., 2020b). Consequently, mechanisms to protect cell components against ROS were evolved and the antioxidant defenses can be induced either by respiratory growth or in the presence of pro-oxidants (Moradas-Ferreira and Costa, 2000). Yeasts have several inducible adaptive stress responses to oxidants regulated at the transcriptional and posttranscriptional levels (Jamieson, 1998; Moradas-Ferreira and Costa, 2000). Stresses commonly arising during industrial fermentations, such as supra-optimal temperatures or presence of inhibitory concentrations of ethanol, acetic acid or lactic acid lead to oxidative stress that occurs when cellular defense mechanisms are unable to cope with existing ROS (Costa and Moradas-Ferreira, 2001; Abbott et al., 2009; Teixeira et al., 2009; Morano et al., 2012; Charoenbhakdi et al., 2016).

The cell wall or, more broadly, the cell envelope, has been associated with oxidative stress toxicity and tolerance. This involvement has been unveiled by studies focused on *S. cerevisiae* exposure to pro-oxidant agents such as hydrogen peroxide, lipid hyperoxides, diamine, catecholamines, and organic hydroperoxides, such as cumene hydroperoxide (CHP) and linoleic acid hydroperoxide (LoaOOH) (Staleva et al., 2004; Vilella et al., 2005; Petkova et al., 2010; Taymaz-Nikerel et al., 2016). Oxidative stress resulting from exposure to these agents induces distinct responses in *S. cerevisiae*. For instance, a quantitative proteomics study reported differences in the activation of the CWI pathway between hydrogen peroxide, CHP and diamide (Pandey et al., 2020). Decreased cell permeability, a property influenced by the thickness and composition of the cell wall and plasma membrane, results in increased resistance to pro-oxidant compounds by limiting their diffusion into the cell (Sousa-Lopes et al., 2004). Furthermore, membrane lipid composition is a determinant of oxidative stress resistance, with cells containing a higher level of saturated fatty acids being more resistant than cells with a higher level of polyunsaturated fatty acids (Jamieson, 1998).

**FIGURE 3:**
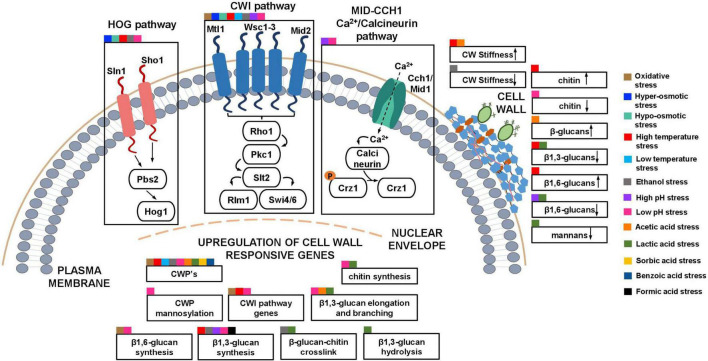
Schematic model of the described *S. cerevisiae* adaptive responses common to multiple industrial relevant stresses involving the cell wall. The cell wall integrity pathway (CWI) and other signaling pathways (HOG and Calcineurin pathway) that, in collaboration with the CWI pathway and the upregulation of cell wall genes, lead to changes in the composition and nanomechanical properties of the cell wall in response to different stresses are highlighted. Squares of different colors indicate the different industrial-relevant stresses for which data is available in the literature.

Diamide induces the formation of disulfide bonds in the three-dimensional structure of CWPs, causing changes in the morphology of the cell outer layer and the increase of cell wall thickness (De Souza Pereira and Geibel, 1999; Vilella et al., 2005). CHP causes oxidative damage to the cell wall periphery leading to the upregulation of genes related to cell wall biogenesis (*HSP150*), CWI pathway regulation (*RHO1, ROM2*) and β1,6-glucan synthesis (*KRE5, KRE6, KHN1*) (Sha et al., 2013; Figure 3). The transcriptional reprogramming of yeast in response to oxidative stress is mainly regulated by two oxidative stress-responsive transcription factors, Yap1 and Skn7, and the general stress transcription factors, Msn2/Msn4 (Auesukaree, 2017). Notably, Skn7 has an important role in the regulation of genes involved in the maintenance of cell wall integrity, including genes coding for CWPs (Alloush et al., 2002; Li et al., 2002; Levin, 2011). The CWI pathway sensors Wsc1, Wsc2, Mtl1, and Mid2 play an important role in the sensing of oxidative stress induced by H_2_O_2_ (Vilella et al., 2005; Jin et al., 2013; Vélez-Segarra et al., 2020). Surviving and overcoming oxidative stress induced by H_2_O_2_, as for diamine, requires Pkc1 and the upstream element of the CWI pathway Rom2 (Vilella et al., 2005; Figure 3).

In *Yarrowia lipolytica*, a promising oleaginous yeast (Ledesma-Amaro and Nicaud, 2016), oxidative stress induced by H_2_O_2_ leads to changes in the morphology of the cell wall, with the formation of globular surface structures in the cell wall surface (Arinbasarova et al., 2018). When exposed to soluble complexes of UO_2,_ the mRNA levels from genes involved in elongation of β1,3-glucan and chitin synthesis were reduced in a tropical marine strain of *Y. lipolytica*, able to immobilize in the cell surface uranium often associated with oxidative stress (Kolhe et al., 2020). These complexes, formed above pH 7.0, are prevalent in aquatic environments such as rivers or sea water, being this strain considered promising for the bioremediation of uranium-contaminated aquatic environments (Kolhe et al., 2020, 2021).

### Osmotic stress

Hyper-osmotic stress is common in industrial bioprocesses (Mattanovich et al., 2004). Very high gravity fermentations are frequently used to enhance product titres in specific sectors, such as in first-generation bioethanol production by increasing initial sugar concentration and, consequently, osmotic pressure (Deparis et al., 2017). In winemaking and baking, yeasts also have to grow in hyper-osmotic environments and their performance depends on the ability to adapt and respond to this stress (Attfield and Kletsas, 2000; Noti et al., 2015). Also, the increase of osmotic pressure induced by the addition of stressful concentrations of sugars or salts is beneficial for food preservation (Gonzalez et al., 2016). In yeasts, the High Osmolarity Glycerol (HOG) and the CWI signaling pathways are central to osmotic stress-induced response and control of cellular turgor (Davenport et al., 1995; Hohmann, 2002a). Although there are many similarities in how the yeast cell responds to osmotic stress caused by different agents, thus allowing the description of a generalized stress response, there are also specific features for each osmolyte or salt stress (Gonzalez et al., 2016).

Sudden exposure to increased osmolarity leads to cell shrinkage due to water efflux and likely causes the release of plasma membrane material, as well as changes in cell wall structure (Morris et al., 1983; Claro et al., 2007; Saldaña et al., 2021). The decrease in cell volume is accompanied by changes in cell morphology and surface roughness (Adya et al., 2006; Saldaña et al., 2021). Intracellularly, a compensatory response is triggered, recruiting water from the vacuole into the cytoplasm (Mager and Siderius, 2002). These changes occur immediately after an osmotic upshift, likely caused by rapid biophysical forces (Ene et al., 2015). To survive such a shock, the properties of the cell wall have to be modified to have enough elasticity to respond to shifts in osmolarity, while maintaining enough rigidity to preserve cell morphology and integrity (Ene et al., 2015). The rapid changes of cellular volume and shape occurring in response to hyperosmotic shock that are fully reversible when cells are introduced in an isosmotic solution, are evidences of the remarkable plasticity of the cell wall (Saldaña et al., 2021).

The HOG pathway (Figures 2, 3) is a MAPK signal transduction system and the major pathway in the adaptation of yeast cells to increased osmolarity (Hohmann, 2002a). The pathway relies on two osmolarity sensors, Sln1 and Sho1. During an osmotic upshift, Sln1 is transiently inhibited, diminishing the levels of phosphorylated Ssk1. Unphosphorylated Ssk1 can then interact with Ssk2/Ssk22, which in turn phosphorylates Pbs2. The Sho1 osmosensor also transmits a signal to Pbs2 via the MAPKKK Ste11 (Hohmann, 2009). The phosphorylated Pbs2 activates Hog1 and modulates the expression of several genes (Hohmann, 2009), including the transcriptional activation of *GPD1* and *GPP2* genes, encoding a glycerol 3-phosphate dehydrogenase and a glycerol-1-phosphatase respectively, involved in glycerol production (Hohmann, 2009). In response to hyper-osmotic stress, Hog1 phosphorylates Rgc2, a regulator of the Fps1 glycerol channel, causing the dissociation of Rgc2 from Fps1 and the consequent glycerol channel closure (Lee et al., 2013). Together, the blockade of glycerol efflux by the closing of the Fps1 channel and the increase in glycerol biosynthetic capacity lead to glycerol accumulation in the cytoplasm and effectively counteract the loss of turgor pressure caused by an osmotic upshift (Jiménez-Gutiérrez et al., 2020b).

A coordinated interplay between the HOG pathway and the CWI pathway allows yeast cells to better adapt to hyper-osmotic stress conditions (García-Rodríguez et al., 2005; Vaz et al., 2020; Figure 3). The CWI pathway appears to be indirectly activated by an increase in turgor pressure caused by the accumulation of glycerol, leading to transient phosphorylation of Slt2 that depends on a functional HOG pathway (García-Rodríguez et al., 2005). Nevertheless, the CWI sensors Mid2 and Wsc1 seem to be involved in the sensing of Hog1-driven accumulation of glycerol (García-Rodríguez et al., 2005). Further evidences of the interplay between the HOG pathway and the CWI pathway were reported (Vaz et al., 2020). Chemogenomic studies have implicated genes related to β1,3-glucan synthesis (*FKS1*), β1,6-glucan synthesis (*KRE6*) and cell wall mannosylation (*MNN10, ANP1*) as determinants of tolerance to high-glucose and sucrose concentrations (Ando et al., 2006; Teixeira et al., 2010), known to induce hyper-osmotic stress (Deparis et al., 2017).

In *Y. lipolytica*, hyper-osmotic stress was also found to lead to cell wall remodeling (Kubiak-Szymendera et al., 2022). Yl*HOG1* deletion impacts filamentous growth, cytokinesis, and resistance to cell wall perturbing agents (Rzechonek et al., 2018). A quantitative proteomic analysis has shown that, among the upregulated proteins under hyper-osmotic stress is Pil1, involved in formation of membrane-associated protein complexes commonly referred as eisosomes and distributed across the cell surface periphery, and *UTR2* coding for a CWP, involved in glucan-chitin crosslinking in *S. cerevisiae* (Kubiak-Szymendera et al., 2022). Other non-*Saccharomyces* species, such as *Zygosaccharomyces rouxii, Debaryomyces hansenii*, and *Pichia sorbitophila* are notable for their osmotolerance (Bubnová et al., 2014). The spoilage yeast of high-sugar or high-salt foods *Z. rouxii* appears to react to hyperosmolarity similarly to *S. cerevisiae*, accumulating intracellular glycerol through the increased expression of Zr*GPS1* and decreased expression of Zr*FPS1* (Pøibylová et al., 2007; Hou et al., 2013; Guo et al., 2020). Among *Z. rouxii* upregulated genes under high osmolarity stress are *FKS1*(encoding a β1,3-glucan synthase), *UTR2* (encoding a cell wall transglycosylase), *KRE9* (encoding a glycoprotein involved in β-glucan assembly), *CHS1* (encoding a chitin synthase) and *KAR2* (encoding an ATPase involved β1,6-glucan synthesis and also involved in the translocation of proteins into the endoplasmic reticulum) (Guo et al., 2020). The upregulation of the encoding genes helps to explain why the cell wall became thicker as the cell volume decreases, resulting in a smaller amplitude of cell size variation (Guo et al., 2020).

In a *Zygosaccharomyces mellis* strain, isolated from honey and tolerant to high-glucose concentrations (Liu et al., 2016, 2021), the genes *KRE5* (involved in β1,6-glucan synthesis) and *SLT2* (encoding a kinase of the CWI pathway), are upregulated under hyper-osmotic stress (Liu et al., 2021). This indicates that, in *Z. mellis*, the maintenance of cell wall integrity under this stress is also important. In *D. hansenii*, the cell wall was also shown to play a critical role in osmosensing and genes involved in CWP mannosylation (*MNN1, PMT2, PSA1*, and *MNT1*) are upregulated in response to hyper-osmotic stress (Thomé, 2007; Gonzalez et al., 2009b).

### Heat stress

Another obstacle that can be faced by yeast cell factories is temperature stress, in particular heat stress. Heat stress is common during alcoholic fermentations to produce alcoholic beverages or bioethanol due to their exothermic nature. If the temperature is not controlled, a significant rise in temperature occurs (Parapouli et al., 2020; Walker and Basso, 2020). Given that in the presence of other stresses, in particular of ethanol or acetic acid stress, the optimum and the maximum temperatures for growth decrease as the stress level increase, even temperatures close to the optimal range of growth can become lethal temperatures, depending on the level of stress (van Uden, 1985; Godinho et al., 2021). Despite the importance of cold stress, the related literature is scarce in the context of this review, so this part of the article is essentially dedicated to heat stress.

At supra-optimal temperatures in the absence of any other stress (higher than 35–37°C), *S. cerevisiae* activates the heat shock response (HSR) and undergoes physiological changes which include membrane and cell wall restructuring (Verghese et al., 2012; Schiavone et al., 2014). During heat stress, β1,6-glucan and chitin content increases by 20 and 100% respectively, and β1,3-glucans levels decrease by 45% (Schiavone et al., 2014; Figure 3). The increased β1,6-glucan synthesis under heat stress is in accordance with a higher number of cross-linkages between this polymer and chitin, presumably compensating cell wall weakening during this stress (Kapteyn et al., 1999a; Schiavone et al., 2014). Heat stress also induces changes in the morphology of the yeast cell surface with the formation of circular structures in the surface of heat-stressed cells (Pillet et al., 2014). The emergence of these structures is, apparently, related to a dysfunction in the budding machinery, accompanied by a concomitant increase in chitin and cell wall stiffness, regulated by the CWI pathway (Pillet et al., 2014; Figure 3) activated in response to heat stress (Torres et al., 1991; Lee and Levin, 1992; Irie et al., 1993). Together with Msn2/4, Hsf1 is a transcription factor responsible for the bulk of the HSR (Imazu and Sakurai, 2005; Truman et al., 2007). Heat stress also promotes glycerol efflux by the opening of Fps1 channels regulated by the CWI pathway (Dunayevich et al., 2018). The resulting turgor loss stimulates the activation of the HOG pathway, promoting glycerol production and the re-establishment of turgor pressure (Dunayevich et al., 2018). Heat shock leads to the intracellular accumulation of trehalose in *S. cerevisiae* (Attfield, 1987; Hottiger et al., 1987). Since trehalose and thermotolerance are closely related, trehalose was suggested to act as a thermoprotectant (Hottiger et al., 1989). The intracellular accumulation of trehalose causes a decrease of the specific growth rate that may trigger the environmental stress response (ESR) and higher thermotolerance (Gibney et al., 2015). The accumulation of trehalose increases cytosolic osmolarity and turgor pressure, mimicking hypotonic stress, and causing plasma membrane stretching known to lead to activation of the CWI pathway (Kamada et al., 1995; Mensonides et al., 2005). Consistently, preventing trehalose synthesis by deletion of the *TPS1* gene led to decreased activation of the CWI pathway upon exposure of yeast cells to heat stress (Mensonides et al., 2005). Interestingly, a recent study suggests that the UDP-Glucose Pyrophosphorylase Ugp1 is required for heat stress response by influencing trehalose and glucan content (Zhang and Xu, 2021).

Although the mechanism behind heat-induced activation of the CWI pathway is not clear, the Wsc1 and Mid2 CWI sensors appear to be involved in the activation of CWI pathway, reinforcing the notion that this stress is ultimately transmitted to the cell surface (Verna et al., 1997; Rajavel et al., 1999; Martín et al., 2000; Levin, 2011; Verghese et al., 2012). In fact, Wsc1 sensors form clusters in the plasma membrane upon heat stress (the so-called Wsc1 sensosomes) enhancing the CWI pathway signaling capability (Heinisch et al., 2010). The expression of the CWI pathway *SLT2* gene, the *HSP150* gene encoding a protein required for cell stability and the CWP-encoding gene *YGP1* are induced in response to heat stress (Causton et al., 2001; Varol et al., 2018; Figure 3). Also, *FKS2*, encoding a β1,3-glucan synthase, is upregulated during heat stress, being its expression regulated by both CWI and Calcineurin pathways (Zhao et al., 1998; Figure 3).

The ability to withstand elevated temperatures while maintaining high growth rates and ethanol productivity can be beneficial for bioethanol production and could alleviate some of the costs associated with the cooling of the bioreactors often required to allow an adequate fermentative performance (Lehnen et al., 2019; Thorwall et al., 2020). Two prominent examples of thermotolerant yeast species, shown to be able to grow relatively well above 40°C, are *Kluyveromyces marxianus* and *Ogataea polymorpha* (formerly *Hansenula polymorpha*) (Lehnen et al., 2019; Thorwall et al., 2020). In *K. marxianus*, high temperatures were shown to lead to upregulation of genes associated with changes in plasma membrane composition (Fu et al., 2019). Very few is reported concerning changes occurring in *K. marxianus* cell wall under heat stress but a strain isolated from agave when grown at 42°C exhibit increased sensitivity to lyticase than when grown at 30°C, indicating that heat stress may affect cell wall integrity (Castillo-Plata et al., 2021). In *Ogataea* species, thermotolerance has been attributed to a structural predisposition of the cell envelope, related with membrane and cell wall composition, allowing a higher cell envelope stability (Lehnen et al., 2019). Among the candidate genes that contribute to heat tolerance in *O. polymorpha* is an ortholog of *S. cerevisiae PSA1*, coding for GPD-mannose pyrophosphorylase, involved in cell wall biosynthesis (Seike et al., 2021). In the genus *Ogataea*, Mpk1 appears to be involved in CWI signaling in response to heat stress, but, different *Ogataea* strains exhibit different growth phenotypes when *MPK1* is absent (Kim et al., 2018). Compared to *O. polymorpha, O. parapolymorpha* has a thinner β-glucan and chitin layer with short mannan chains and the derived deletion mutant *mpk1*Δ exhibit more severe growth defects during heat stress and higher susceptibility to cell wall-perturbing agents, leading the authors to consider these differences related to cell wall structural differences (Kim et al., 2018).

The characterization of the yeast response to low temperatures is also important in industry-related bioprocesses (Liszkowska and Berlowska, 2021), in particular in many wine and beer fermentations, resulting in the retention of more volatile compounds that influence the sensory properties of the product (Liszkowska and Berlowska, 2021). Furthermore, cold-adapted spoilage yeasts can potentially impose health risks to the consumers and economic burden in the Food industry, being able to grow and proliferate at temperatures at which food products are refrigerated (Fleet, 2011). Among the genes that are upregulated in response to cold temperatures are genes encoding cell wall mannoproteins (*TIR1-2, TIR4, TIP1*, and *DAN1*) (Abramova et al., 2001; Sahara et al., 2002; Homma et al., 2003; Schade et al., 2004; Murata et al., 2006; Abe, 2007; Figure 3). Increased resistance to the cell wall-perturbing compound SDS in cold-shock-stress-induced cells has also been attributed, at least partially, to the upregulation of *DAN1* (Abe, 2007). The CWI pathway *WSC1, BCK1*, and *SLT2* genes encoding a CWI sensor, the MAPKKK Bck1 and the MAPK Slt2, respectively, have also been implicated in response and adaptation to cold temperature-induced-stress, being Slt2 phosphorylation partially dependent on the Wsc1 CWI sensor (Córcoles-Sáez et al., 2012; Figure 3). Psychrophilic yeasts, yeasts adapted to low temperatures, with an optimal growth performance at 15°C, are studied due to its biotechnological potential, in particular for the production of cold-active enzymes (Buzzini et al., 2012). Interestingly, the absence of the *SWI4* or *SWI6* genes, encoding transcriptional activators of the CWI pathway in *Metschnikowia australis* W7-5, leads to impaired growth at temperatures as low as 5°C, suggesting that these genes are determinants of tolerance to low temperature in this species (Wei et al., 2021).

### Ethanol – and methanol – induced stresses

Ethanol toxicity is arguably the major environmental stress limiting industrial titres and overall productivity in a wide range of industrial yeast fermentations (Gibson et al., 2007; Parapouli et al., 2020). Accumulation of high concentrations of ethanol, whether during bioethanol production, wine and brewing industries, or other alcoholic fermentations, often results in decreased fermentation yield or complete cessation of yeast metabolic activity and fermentation arrest (Walker and Basso, 2020).

Due to its liposolubility, ethanol disrupts plasma membrane lipid organization, increasing its fluidity, non-specific permeability, and compromising transmembrane electrochemical potential (van Uden, 1985). Among the adaptive responses is the alteration of plasma membrane composition (e.g., increase in ergosterol content and in unsaturated/saturated fatty acid ratio) leading to increased lipid order and counteracting plasma membrane fluidisation (Navarro-Tapia et al., 2018), together with and adaptive response at the level of the cell wall (Ding et al., 2009; Stanley et al., 2010). Remarkably, the impairment of plasma membrane integrity was shown to influence the nanomechanical properties of the cell wall during ethanol stress (Schiavone et al., 2016) (Figure 3). Specifically, ethanol-induced changes to the plasma membrane, compromising the proper delivery of plasma membrane-anchored GPI-CWPs to the membrane via the secretory pathway, affect their crosslinking to cell wall polysaccharides resulting in reduced cell wall stiffness (Schiavone et al., 2016; Figure 3).

Several chemogenomic studies have implicated genes related to cell wall biosynthesis, cell wall remodeling, and CWI pathway as determinants of ethanol tolerance (Kubota et al., 2004; Fujita et al., 2006; van Voorst et al., 2006; Auesukaree et al., 2009; Teixeira et al., 2009). Additionally, suitable supplementation of the fermentation medium with potassium, zinc or inositol was shown to improve tolerance to ethanol stress (Krause et al., 2007; Zhao et al., 2009; Xu et al., 2020). Genes reported as determinants of ethanol tolerance include *MNN10, MNN11, ANP1*, and *HOC1*, encoding four subunits of the polymerase complex responsible for the elongation of the mannose backbone present in CWPs, and *LDB7*, encoding a component of the chromatin structure remodeling complex, involved in the regulation of the mannosylphosphate content of mannoproteins (Kubota et al., 2004; Auesukaree et al., 2009; Teixeira et al., 2009). Moreover, the CWP-encoding genes *PIR3, SED1, SPI1*, and *YGP1*, as well as the GPI-CWPs-encoding genes of the *TIR* family (*TIR1-3* and *TIP1*), were found to be transcriptionally responsive to ethanol stress (Ogawa et al., 2000; Rossignol et al., 2003; Wu et al., 2006; Udom et al., 2019; Figure 3). The overrepresentation of genes related to the mannoprotein-rich outer layer of the cell wall in response to ethanol-induced stress suggests that mannoproteins have a role in yeast adaptation to this stress, limiting ethanol’s access to the plasma membrane and thus counteracting ethanol-induced membrane permeabilization and subsequent deleterious effects. Due to its amphiphilic nature, ethanol can bind to the exposed proteins at the outer layer of the cell wall, likely altering its organization and increasing cell wall porosity (Zlotnik et al., 1984; De Nobel et al., 1990; Udom et al., 2019).

Genes related to chitin and glucan synthesis, namely *CHS1* (chitin), *FKS1* (β1,3-glucans), *ACF2* (β1,3-glucans), and *KRE6* (β1,6-glucans), as well as genes involved in cell wall integrity (*PUN1*) and cell wall organization (*SIT4*), were also found to be involved in ethanol tolerance (Kubota et al., 2004; Auesukaree et al., 2009; Teixeira et al., 2009; Mota et al., 2021). The upregulation of *FKS1* and also of *SED1* and *SMI1* genes, involved in cell wall biosynthesis depends on the Znf1 transcription factor, recently implicated in adaptation to ethanol stress (Samakkarn et al., 2021). Consistent with a transcriptional response involving multiple components of the cell wall under ethanol stress, elements of the CWI signaling pathway, namely the membrane sensors Mid2 and Wsc1, the MAPKKK Bck1, and the MAPK Slt2, were found to be crucial for maximum tolerance to ethanol stress (Fujita et al., 2006; Auesukaree et al., 2009; Teixeira et al., 2009). Also, yeasts deficient in either one of the two Slt2-activated SBF subunits, Swi4 and Swi6, reveal higher sensitivity to ethanol (Udom et al., 2019). The SBF complex controls the expression of *FKS2* and *CRH1*, coding for a β1,3-glucan synthase and a chitin transglycosylase involved in glucan-chitin crosslinking, respectively; these genes were shown to be upregulated during ethanol stress (Udom et al., 2019; Figure 3). Additionally, the HOG pathway also seems to contribute to the regulation of the transcriptional response of cell wall genes during ethanol stress, since the dysfunction of the HOG pathway leads to decreased expression of *FKS2* and *CRH1* (Udom et al., 2019). Furthermore, the wall of yeast cells exposed to ethanol exhibits higher resistance to lyticase and zymolyase treatment (Udom et al., 2019). In summary, the mechanism underlying ethanol tolerance involves a collaborative role of the CWI and HOG signaling pathway in the transcriptional regulation of cell wall genes leading to cell wall changes (Udom et al., 2019; Figure 3).

Not many non-conventional yeasts can compete with *S. cerevisiae* when it comes to ethanol production and tolerance, but some have other interesting features that could make their use in the bioethanol industry appealing. *K. marxianus*, known for its thermotolerance and ability to metabolize several carbon sources (Thorwall et al., 2020), has a fair number of studies dedicated to understanding the response to ethanol stress (Diniz et al., 2017; Alvim et al., 2019; Mo et al., 2019; da Silveira et al., 2020), although not much is known about ethanol-induced changes to its cell wall. However, the adaptive evolution under ethanol stress was found to lead to the improvement of multiple pathways, including cell wall biogenesis, suggesting that cell wall remodeling is part of a strategy to mitigate the toxic effects of ethanol, also in this species (Mo et al., 2019). The increase in cell wall thickness also occurs in *Saccharomyces boulardii* under ethanol stress (Ramírez-Cota et al., 2021). *S. boulardii* is known for its probiotic capacities as a biotherapeutic agent in infections and medical disorders (Hudson et al., 2016; Suvarna et al., 2018; Hossain et al., 2020) and is a promising species to be used in certain crafts of beer fermentation (Ramírez-Cota et al., 2021). In *Issatchenkia orientalis*, with potential to be used in winemaking, cell wall-related genes *GAS4* (β-1,3-glucanosyltransferase involved in glucan elongation), *FLO1* (CWP involved in adhesion events important for flocculation) and *IFF6* (GPI-CWP involved in cell wall organization) were found to be up-regulated under ethanol stress (Miao et al., 2018).

A recent chemogenomic analysis reported that, in *S. cerevisiae*, methanol and ethanol, share genetic determinants of tolerance involved in cell wall maintenance (Mota et al., 2021). Methanol is a feedstock alternative to sugar-based raw substrates for biorefinery processes and a toxic compound commonly found in crude glycerol, a by-product of the biodiesel industry, and in hydrolysates from pectin-rich biomass residues (Yasokawa et al., 2010; Martins et al., 2020; Mota et al., 2021). Methanol and ethanol tolerance genes include *FKS1* and *SMI1* (β1,3-glucan synthesis), *ROT2* (β1,6-glucan synthesis), *MNN11* (mannosylation of CWPs), and *WSC1* (CWI pathway membrane sensor). However, *KRE6, CWH41* (β1,6-glucan synthesis) and *GAS1* (β1,3-glucan chain elongation and branching) were found to be required for maximum tolerance to methanol but not for equivalent inhibitory concentrations of ethanol (Mota et al., 2021). This suggests that, despite the similarities of these alcohols, equivalent inhibitory concentrations might impact the cell wall differently and, as such, elicit not fully overlapping remodeling responses (Mota et al., 2021). The CWI pathway is implicated in methanol adaptation in *Komagataella phaffii* (formerly *Pichia pastoris*), involving the upregulation of the *SLT2* homolog encoding gene in *K. phaffii* (Zhang et al., 2020). The homolog of Wsc1 and Wsc3 CWI sensors in the methylotrophic yeast *K. phaffii* were implicated in sensing methanol, interacting upstream with the *K. phaffii* homolog of Rom2, a GEF of *S. cerevisiae* CWI pathway for activation of methanol-inducible genes (Ohsawa et al., 2017). Additionally, differences in methanol metabolism, vector transformation efficiency, growth and heterologous protein production between different *K. phaffii* strains were related with cell wall integrity (Zhang et al., 2020), providing another example of the important role of cell wall.

### Low- or high-pH-induced stress

Cell wall composition and architecture and the CWI pathway were also implicated in yeast adaptive response to acid and alkaline stress conditions (Claret et al., 2005; Serrano et al., 2006; De Lucena et al., 2012). However, the mechanisms underlying yeast response and tolerance to acidic conditions are complex and dependent not only on the pH value but also on the nature of the acid used to adjust low pH. Strong inorganic acids, such as sulphuric acid and hydrochloric acid, are fully dissociated at any external pH, while weak organic acids dissociation depends on medium pH and their pKa, the toxic form being the liposoluble non dissociated form (Carmelo et al., 1996, 1997; Booth and Stratford, 2003). Since the plasma membrane of unstressed cells is very poorly permeable to H^+^, the effect of strong acids relies, essentially, on the concentration of H^+^/medium pH (Carmelo et al., 1996, 1997; Lucena et al., 2020). For this reason, pH stress and stress induced by organic acid stress at low pH are discussed separately.

#### Acid pH-induced stress

In bioethanol production, yeast biomass is reused after being washed between successive batches using inorganic acids. It is a common procedure, frequently carried out at a pH below 3.0 as a means of eliminating contaminant bacteria from pitching yeast. This disinfection treatment, together with the presence of toxic metabolites and other stressful conditions occurring during fermentation, may lead to the loss of cell viability and limit fermentation yield (Simpson and Hammond, 1989; De Melo et al., 2010; Lucena et al., 2020). A scalable and economic solution to control bacterial contamination during alcoholic fermentation is to run the fermentations at low pH (<4.0), at which growth and viability of most bacteria are drastically reduced (Narayanan et al., 2016). Thus, understanding how yeast strains tolerate low pH set up with strong acids may enable the improvement of ethanol yield and reduce production costs (Brosnan et al., 2000). It is likely that during fermentation, organic acids (weak acids) may also play a role since they are produced during yeast metabolism and may be already present in the fermentation medium (e.g., when lignocellulosic biomass hydrolysates are used).

In response to acid pH, the cell wall structure and composition suffers alteration (Cabib et al., 1989; Kapteyn et al., 2001) leading to deformation of surface morphology (De Lucena et al., 2015). Cell wall chitin levels decrease at growth pH values below 5.0, likely as a result of increased chitinase activity (Cabib et al., 1989; Figure 3). Many of the genes found to be upregulated at low pH are related to cell wall biogenesis, including *FKS1* (β1,3-glucan synthase), *GAS1* (β-1,3-glucanosyltransferase involved in cell wall remodeling-elongation of (1 → 3)-β-D-glucan chains and branching), *CHS1* (chitin synthase), *NCW2* (GPI-protein involved in chitin-glucan assembly), *KRE6* (glucosyl hydrolase required for β1,6-glucan synthesis) and *MNN9* (mannosyltransferase subunit involved in wall protein mannosylation) (De Melo et al., 2010; De Lucena et al., 2012; Figure 3). Interestingly, using QTL mapping to uncover the genetic basis of a bioethanol industrial strain Pedra-2 (PE-2) tolerance, a prevalent non-synonymous mutation (A631G) in *GAS1* was identified during growth at low pH induced by sulfuric acid exposure, reinforcing the idea of the relevant role of this GPI-protein in yeast tolerance in acidic environments (Coradini et al., 2021). Together with the up-regulation of genes related with 1,3-β-glucan synthesis, elongation, and anchoring, low pH stress leads to increased cell wall resistance to compounds with β1,3-glucanase activity and to the establishment of more alkali-sensitive linkages between CWPs and the β1,3-glucan network (Kapteyn et al., 2001; De Melo et al., 2010; De Lucena et al., 2012, 2015; Lucena et al., 2020). This suggests that low pH established by a strong inorganic acid affects the β-glucan fraction of the cell wall.

As for other stresses, response to acid pH imposed by inorganic acids involves a crosstalk between different signaling pathways. The CWI signaling pathway is involved in response to low pH in *S. cerevisiae* and has been proposed as the main mechanism for tolerance to acid pH (Claret et al., 2005; De Lucena et al., 2012; Figure 3). Its activation is mainly mediated by Mid2, but also by Wsc1, with the latter appearing to have a more prominent role in activating the general response of the cell to this stress (Claret et al., 2005; De Lucena et al., 2012). The proposed model is that cell wall injury due to acid stress results in lower turgor and consequently mimics the effect of hyper-osmotic shock, justifying the activation of the HOG pathway (De Lucena et al., 2012; Figure 3). The HOG pathway then appears to have a dual role. First, Hog1 activates the protein complex Msn2/4, which induces the expression of ESR genes, including *RGD1*, a major regulator of yeast survival at low pH stress, that encodes a protein implicated in the activation of CWI pathway under acid stress (Claret et al., 2005). Second, the Hog1 kinase may help to establish a positive feedback loop at the downstream module of the CWI pathway by cooperating with the Slt2-activated Rlm1 transcription factor to increase the expression of *SLT2* (Claret et al., 2005; De Lucena et al., 2012). Therefore, the HOG pathway can activate the CWI pathway while bypassing its membrane sensors. The Ca^2+^/calmodulin-dependent calcineurin pathway is also involved in the response to acid pH, and interacts with the CWI pathway by activating Cch1/Mid1 calcium channels by Slt2, the Crz1 transcription factor by Rho1-Skn7, and through the cooperation between Slt2 and Crz1 for the expression of *FKS2* in response to cell wall injury (De Lucena et al., 2012; Figure 3). This Ca^2+^-dependent response is likely responsible for the increment of CWI protein trafficking and their localisation at cell surface to repair the structural changes caused by medium acidification (Lucena et al., 2020). Together, CWI, HOG, and calcineurin signaling pathways ensure the post-translational activation of the transcription factors needed to promote cell wall maintenance and regeneration to survive acidic pH stress.

#### Alkaline pH stress

The yeast *S. cerevisiae* proliferates better at acidic than at neutral or alkaline pH and medium alkalinisation has widespread effects in yeast physiology (Serra-Cardona et al., 2015). An increase of pH from 4.0 to 6.0 leads to the decrease of the relative proportion of β1,6-glucans in the β-glucan fraction of the cell wall (Aguilar-Uscanga and François, 2003) and cells grown at pH 6.0 are more susceptible to zymolyase treatment (Aguilar-Uscanga and François, 2003; Figure 3). The CWI pathway is necessary for tolerance to alkaline pH, as shown by the strong alkali-sensitive phenotype of the *bck1*Δ, *slt2*Δ, *swi4*Δ, and *swi6*Δ mutants (Serrano et al., 2006). The alkaline stress-mediated activation of Slt2 was also shown to depend on the CWI Wsc1 membrane sensor (Serrano et al., 2006; Kwon et al., 2016; Figure 3). Both *FKS2* and *GAS1* encoded proteins, involved in the synthesis and elongation of β1,3-glucans respectively, are required to resist alkaline stress, and likely play a major role in altering the ratio between different types of glucans in the cell wall (Serrano et al., 2006). The Ca^2+^-dependent calcineurin response has also been implicated in the regulation of cell wall synthesis during alkaline stress by the upregulation of *FKS2* expression via the calcineurin-activated transcription factor Crz1 (Viladevall et al., 2004; Figure 3).

Changes in cell wall composition were also implicated in *Y. lipolytica* adaptation to high pH stress (Sekova et al., 2019). In particular, the structural mannoprotein YlPir1 is abundant in the cell wall in unstressed conditions but absent when *Y. lipolytica* cells are exposed to high pH stress (Sekova et al., 2019). This readjustment is consistent with the fact that mannans, unlike other main polysaccharides of the cell wall, are prone to alkaline hydrolysis, and therefore unstable at high pH (Sekova et al., 2019).

To summarize, there are major differences between the signaling responses elicited by acidic and alkaline pH stresses involving the cell wall. First, Mid2 appears to be the main sensor activating the CWI pathway in response to acidic pH, while Wsc1 is the main sensor in alkaline pH (Claret et al., 2005; Fuchs and Mylonakis, 2009; Serra-Cardona et al., 2015). Second, acidic pH stress mostly leads to the transcription of Rlm1-dependent genes, while alkaline pH stress favors transcription of SBF-dependent genes. Third, while the CWI pathway manages acidic stress in a Hog1-dependent manner, response to alkaline stress is Hog1-independent (Fuchs and Mylonakis, 2009).

### Organic acid-induced stress

The response and tolerance of the yeast cell to the various industrially relevant weak acids and the underlying toxicity mechanisms are not fully shared by all the acids, with specific mechanisms for a weak acid/group of weak acids (Mira et al., 2010c). In general, and broadly speaking, the higher the lipophilicity of each of a weak acid is, the higher its toxicity. The straight medium chain weak acids (e.g., butyric, hexanoic, octanoic, and decanoic acids) and sorbic and benzoic acids are more lipophilic and toxic than the short-chain volatile fatty acids (VFA) formic, acetic, and propionic acids (Mira et al., 2010c; Skoneczny, 2018).

#### Acetic acid stress

Acetic acid is widely used as a food preservative in the food industry and is also a major inhibitory compound present in lignocellulosic biomass hydrolysates limiting the use of this low cost and abundant biomass (Palma et al., 2018; Cunha et al., 2019). Acetic acid is also produced by yeast metabolic activity and can lead, together with ethanol and other yeast toxic metabolites, to decreased ethanol yield and even fermentation arrest depending on the level of stress (Palma et al., 2018; Cunha et al., 2019; Palma and Sá-Correia, 2019). Elucidation of the mechanisms underlying yeast adaptation and tolerance to acetic acid is instrumental to pave way for strain and process optimisation in several important biotechnological and food industries.

When the external pH is below acetic acid pK_*a*_ (below 4.75 at 25°C) (Lide, 2003), the undissociated form of the acid (CH_3_COOH) is able to passively diffuse through the plasma membrane lipid bilayer (Casal et al., 1996; Mira et al., 2010c; Palma et al., 2018; Palma and Sá-Correia, 2019). Once in the near-neutral cytosol, acetic acid dissociates into the negatively charged acetate counterion, CH_3_COO^–^, releasing protons, H^+^. Being unable to cross the hydrophobic lipid layer due to the electric charge, these ions accumulate in the cytosol, resulting in decreased intracellular pH, increased turgor pressure and oxidative stress, disrupting normal metabolism (Mira et al., 2010c; Palma et al., 2018; Palma and Sá-Correia, 2019; Ribeiro et al., 2022).

Several genome-wide studies have sought to shed light into the global mechanisms involved in the response and tolerance of *S. cerevisiae* to acetic acid (Kawahata et al., 2006; Abbott et al., 2007; Almeida et al., 2009; Mira et al., 2010a,b; Bajwa et al., 2013; Longo et al., 2015). Increased cell wall impermeabilization in adapted yeast cells can reduce the passive diffusion of the weak acids into the cytosol, in this way restraining the futile cycle associated with the re-entry of the liposoluble acid form after the active expulsion of its counter-ion from the cell interior (Ullah et al., 2013). Recently, it was reported a coordinate and comprehensive view on the time course of the alterations occurring at the level of the cell wall during adaptation of a yeast cell population to sudden exposure to a sub-lethal stress induced by acetic acid (Ribeiro et al., 2021). Yeast cell wall resistance to lyticase activity was found to increase during acetic acid-induced growth latency, corresponding to the period of yeast population adaptation to sudden exposure to acetic acid. This response was correlated with the increase of cell stiffness, assessed by atomic force microscopy (Ribeiro et al., 2021; Figure 3). The increased content of cell wall β-glucans, also assessed by fluorescence microscopy, and the slight increase of the transcription level of the *GAS1* gene encoding a β-1,3-glucanosyltransferase that leads to elongation of β1,3-glucan chains, were also implicated (Ribeiro et al., 2021; Figure 3). These observations reinforce the notion that the adaptive yeast response to acetic acid stress involves a coordinate alteration of the cell wall at the biophysical and molecular levels, essential to limit the futile cycle associated to the re-entry of the toxic acid form after the active expulsion of acetate from the cell interior (Ribeiro et al., 2021).

The adaptive genomic response to acetic acid in *S. cerevisiae* is mainly regulated by the Haa1 transcription factor involved in the direct, or indirect, transcriptional activation of approximately 80% of acetic acid-responsive genes and likely involved in the response at the cell wall level (Mira et al., 2010a, 2011). Haa1 increased expression or Haa1 specific mutations lead to increased tolerance to acetic acid stress and to a lower intracellular accumulation of acetate (Tanaka et al., 2012; Palma et al., 2018). Overexpression of *HAA1* improves cell wall robustness in response to this weak acid, as suggested by the decreased susceptibility of the cell wall to lyticase activity mediated disruption (Cunha et al., 2018). Also, *YGP1* and *SPI1*, encoding CWPs, belong to the Haa1 regulon; they are upregulated under acetic acid stress and contribute to yeast tolerance (Simões et al., 2006; Sakihama et al., 2015; Figure 3). Increased mRNA levels from *YGP1* were reported in cells overexpressing *HAA1* (Tanaka et al., 2012). These results suggest that not only the activation of acetate expulsion through efflux pumps is involved in acetic acid tolerance, as proposed (Piper et al., 1998; Holyoak et al., 1999; Tenreiro et al., 2002; Fernandes et al., 2005; Kawahata et al., 2006; Mira et al., 2010b), but a more efficient restriction of the diffusional entry of acetic acid, partially dependent of the CWP Ygp1, can also be involved (Tanaka et al., 2012). The Znf1 transcription factor was also implicated in acetic acid tolerance and in the upregulation of the *YGP1* gene (Songdech et al., 2020).

Genes involved in CWP mannosylation (*MNN2, MNN9, MNN11, KTR4*), chitin synthesis (*CHS1, CHS5*), β1,3-glucan synthesis (*FKS1, ROM2*), and β1,6-glucan synthesis (*KRE6*) were also reported as being required for maximum tolerance of *S. cerevisiae* to acetic acid stress (Mollapour et al., 2009; Mira et al., 2010b). However, the mRNA levels from *RLM1*, encoding a major transcriptional regulator of the CWI pathway, and from Rlm1-target genes, were found to decrease in cells exposed to acetic acid stress, suggesting that the CWI pathway is not the major key player in acetic acid stress response (Ribeiro et al., 2021).

*Zygosaccharomyces bailii* is a common food spoilage yeast capable of adapting and proliferating in the presence of remarkably high concentrations of acetic acid (Palma et al., 2018). During exposure to acetic acid, several genes involved in the modulation of plasma membrane composition and cell wall architecture were found to be differentially expressed (Antunes et al., 2018). Among those genes is the homologue of *S. cerevisiae YGP1*, encoding a cell wall-related glycoprotein, whose upregulation in the presence of acetic acid was shown to depend on ZbHaa1 (Palma et al., 2017; Antunes et al., 2018), showing a high degree of similarity between the responses involving the cell wall in both yeast species. In a *K. marxianus* strain isolated from agave, lyticase assays showed that the addition of KCl/KOH leads to the increase of cell wall robustness in cells grown in the presence of acetic acid (Castillo-Plata et al., 2021). However, further studies are necessary to elucidate the link between changes in cell wall during acetic acid adaptation and potassium homeostasis (Castillo-Plata et al., 2021). In *I. orientalis*, the expression of *MPG1* gene, coding for a GDP-mannose pyrophosphorylase involved in cell wall synthesis, was also found to be upregulated under acetic acid stress (Li et al., 2021).

#### Lactic acid stress

Lactic acid is another important weak acid in the food industry and also in the pharmaceutical and cosmetic industries. Its industrial production is currently carried out by lactic acid bacteria (Van Maris et al., 2004; Sauer et al., 2010) but bacteria are sensitive to low pH, requiring large amounts of neutralizing agents to counteract the acidification of the fermentation media, thus compromising the recovery yield of precipitated lactic acid (Altaf et al., 2007; Yen et al., 2010). Yeasts typically fare better than bacteria in acidic environments, which has motivated attempts to produce lactic acid through the heterologous expression of lactate dehydrogenases in yeast (Dequin and Barre, 1994; Porro et al., 1999; Pacheco et al., 2012). The success of such strategy and its industrial application require knowledge of the mechanisms behind yeast tolerance to lactic acid stress. Exposure to lactic acid leads to a decrease in cell wall glucan content and, to a lesser extent, of the mannan content of *S. cerevisiae* cell wall (Berterame et al., 2016; Figure 3). Several genes involved in the synthesis of cell wall polysaccharides, cell wall remodeling, and synthesis of mannoproteins are transcriptionally responsive and/or are determinants of tolerance to lactic acid stress (Kawahata et al., 2006; Abbott et al., 2007; Suzuki et al., 2012). Specifically, genes required for glucan remodeling (*EXG1, GAS2, SCW10*), cross-linking between β-glucans and chitin (*CRH1*), chitin synthesis (*CHS1*), and genes encoding mannoproteins required for cell wall stability (*HSP150, PIR3, SED1*), are up-regulated in response to lactic acid stress (Kawahata et al., 2006; Figure 3). Genes encoding elements of the MAPK module of the CWI pathway (*BCK1, SLT2*), as well as genes involved in glucan synthesis (*KRE1, KRE11*) and remodeling (*GAS1*), have also been implicated in lactic acid tolerance (Kawahata et al., 2006; Suzuki et al., 2012).

The Haa1 transcription factor also plays an important role in tolerance to lactic acid and is involved in the control of the expression of CWPs (Abbott et al., 2008; Mira et al., 2010c; Sugiyama et al., 2014). As found for acetic acid stress (Kim et al., 2019), exposure to lactic acid leads to Haa1 translocation from the cytoplasm to the nucleus where gene transcription, in particular of *YGP1* and *SPI1* occurs (Sugiyama et al., 2014). The overexpression of these two genes, encoding CWP, likely confers a stronger protective effect against lactic acid-induced toxicity (Sugiyama et al., 2014).

Due to the high tolerance of *Zygosaccharomyces parabailii* to high lactic acid concentrations at low pH, this species was proposed as a promising novel host for lactic acid production (Ortiz-Merino et al., 2018). In *Z. parabailii*, several cell wall-related genes were found to be down-regulated in the presence of lactic acid (Ortiz-Merino et al., 2018). Under the stress conditions tested, during exponential growth in the presence of lactic acid, a slight decrease in glucans was reported in *S. cerevisiae* and *Z. bailii*, and a slight decrease in mannans in *S. cerevisiae* (Berterame et al., 2016; Kuanyshev et al., 2016).

#### Other weak acids

Propionic acid is commonly used to preserve baked goods and dairy (Suhr and Nielsen, 2004). Transcriptomic and chemogenomic studies have hinted at a role for the cell wall in yeast adaptation to this weak acid (Fernandes et al., 2005; Mira et al., 2009). Specifically, *CWP1* (encoding a GPI-CWP), *BAG7* (β1,3-glucan synthesis), and *KNH1* (β1,6-glucan synthesis) are required to resist propionic acid stress, all of which are regulated by the transcription factor Rim101 (Mira et al., 2009), suggesting that cell wall remodeling during adaptation to propionic acid stress may be dependent on *RIM101* expression.

Sorbic and benzoic acids are two other weak acids used to preserve foods and beverages. In both cases, the *S. cerevisiae* cell wall has been implicated in stress tolerance (De Nobel et al., 2001; Simões et al., 2006). Common to the responses to sorbic and benzoic acids is the induction of *SPI1*, a GPI-CWP that likely leads to the decrease of cell wall porosity and, in turn, limits the access to plasma membrane, thus reducing membrane damage, intracellular acidification, and viability loss (De Nobel et al., 2001; Simões et al., 2006; Figure 3). As previously mentioned, *SPI1* is also activated in response to other weak acids and ethanol stress (Ogawa et al., 2000; Simões et al., 2006; Wu et al., 2006).

Formic acid in an inhibitory weak acid present in lignocellulosic hydrolysates negatively impacting lignocellulosic-based biorefining (Cunha et al., 2019).

Under formic acid stress, *S. cerevisiae* cells exhibited a deformed shape, with collapsed cell wall edges, indicating that the cell wall was damaged, and an FTIR analysis suggested that chitin structure was altered (Zeng et al., 2022). *S. cerevisiae* genes involved in β1,3-glucan synthesis (*FKS1, ELO2*), β1,6-glucan synthesis (*TRS65*), chitin synthesis (*CHS5*), CWP mannosylation (*PMT2*), cell wall integrity (*PUN1*), and CWI pathway regulation (*ROM2*) are important determinants of formic acid tolerance while *FKS3*, encoding an *FKS1-2* homolog, is upregulated in response to this acid stress (Henriques et al., 2017; Zeng et al., 2022; Figure 3). Moreover, the upregulation of *EXG2* encoding a major exoglucanase, and *PKC1*, encoding a CWI pathway kinase, was reported in an industrial *S. cerevisiae* strain (S6), engineered to ferment xylose, when grown in a medium with glucose and xylose supplemented with formic acid (Li et al., 2020).

### Adaptive responses to several industrially relevant stresses involving the cell wall

Some of the responses involving the modification cell wall metabolism and cell wall physicochemical properties in yeasts are shared by relevant industrial stresses (Figure 3). For example, the CWI pathway is implicated in oxidative-, osmotic-, heat and cold-, ethanol- and low and high pH- induced stresses (Kamada et al., 1995; Hohmann, 2002a; Claret et al., 2005; García-Rodríguez et al., 2005; Mensonides et al., 2005; Vilella et al., 2005; Fujita et al., 2006; Serrano et al., 2006; Auesukaree et al., 2009; Teixeira et al., 2009; Córcoles-Sáez et al., 2012; De Lucena et al., 2012; Pillet et al., 2014; Kwon et al., 2016; Udom et al., 2019; Vaz et al., 2020). Also, the coordinated regulation involving the CWI and the HOG pathways occurs during osmotic, heat, ethanol and low pH stresses (Kamada et al., 1995; Hohmann, 2002a; García-Rodríguez et al., 2005; Mensonides et al., 2005; De Lucena et al., 2012; Dunayevich et al., 2018; Udom et al., 2019; Vaz et al., 2020) and the Calcineurin pathway interacts with the CWI pathway during high and low pH stresses (Viladevall et al., 2004; De Lucena et al., 2012). The reported upregulation of genes encoding CWPs was also observed under several stress induced conditions (e.g., under ethanol stress in *S. cerevisiae* and *I. orientalis* and under acetic acid stress in *S. cerevisiae* and *Z. bailii* (Ogawa et al., 2000; Rossignol et al., 2003; Simões et al., 2006; Wu et al., 2006; Sakihama et al., 2015; Palma et al., 2017; Antunes et al., 2018; Miao et al., 2018; Udom et al., 2019) and under oxidative, high and low temperature, low pH, lactic, sorbic and benzoic acids-induced stresses in *S. cerevisiae*) (Abramova et al., 2001; Causton et al., 2001; De Nobel et al., 2001; Sahara et al., 2002; Homma et al., 2003; Schade et al., 2004; Murata et al., 2006; Simões et al., 2006; Abe, 2007; Sha et al., 2013; Sugiyama et al., 2014; Varol et al., 2018). This common response is consistent with the important role of CWP in decreasing cell surface porosity and increasing cell wall stability when coping with stress. The upregulation of genes involved in β1,3-glucan synthesis was also reported to be shared by several stresses (heat, ethanol, low and high pH and formic acid stress) and during hyper-osmotic stress in *Z. rouxii* (Zhao et al., 1998; Viladevall et al., 2004; De Melo et al., 2010; De Lucena et al., 2012; Udom et al., 2019; Guo et al., 2020; Samakkarn et al., 2021; Zeng et al., 2022). Genes involved in β1,3-glucan elongation and branching were also found to be upregulated in response to low pH, acetic and lactic acids-induced stresses and under ethanol stress in *I. orientalis* (Kawahata et al., 2006; De Melo et al., 2010; De Lucena et al., 2012; Miao et al., 2018; Ribeiro et al., 2021). Genes involved in chitin synthesis found to be upregulated were also found to be shared by the response to low pH and lactic acid stress in *S. cerevisiae*, and to hyper-osmotic stress in *Z. rouxii* (Kawahata et al., 2006; De Melo et al., 2010; De Lucena et al., 2012; Guo et al., 2020). Genes involved in β-glucan-chitin crosslinking were also found to be upregulated under ethanol and lactic acid stresses in *S. cerevisiae*, and under hyper-osmotic stress in *Z. rouxii* (Kawahata et al., 2006; Udom et al., 2019; Guo et al., 2020). The upregulation of genes involved in β1,6-glucan synthesis are shared by oxidative and low pH stress in *S. cerevisiae*, and by hyper-osmotic stress in *Z. rouxii* and *Z. mellis* (De Melo et al., 2010; De Lucena et al., 2012; Sha et al., 2013; Guo et al., 2020; Liu et al., 2021). Genes involved in CWI pathway were found to be upregulated in oxidative-, heat- and low pH-induced stress in *S. cerevisiae*, under hyper-osmotic stress in *Z. mellis*, and under methanol stress in *K. phaffii* (Claret et al., 2005; De Lucena et al., 2012; Sha et al., 2013; Varol et al., 2018; Zhang et al., 2020; Liu et al., 2021). Genes involved in CWP mannosylation were found to be upregulated in *S. cerevisiae* under low pH stress and in *D. hansenii* under hyper-osmotic stress (Thomé, 2007; Gonzalez et al., 2009b; De Melo et al., 2010; De Lucena et al., 2012). Genes involved in β1,3-glucan hydrolysis were found to be upregulated under lactic acid stress in *S. cerevisiae* (Kawahata et al., 2006).

Of all the industrially relevant stresses herein described, exposure to several stresses were found to decrease, at least, one type of cell wall polysaccharide in *S. cerevisiae*. Specifically, a decrease in the β-glucan content was reported for heat- (β1,3-glucans), high pH- (β1,6-glucans), lactic acid- (β1,3 and β1,6-glucans) induced stresses, and a decrease in mannans was reported during lactic acid stress (Aguilar-Uscanga and François, 2003; Schiavone et al., 2014; Berterame et al., 2016). A slight decrease in the β-glucan content (β1,3 and β1,6-glucans) was also reported for *Z. bailii* during lactic acid stress (Kuanyshev et al., 2016). A decrease in the chitin content was reported for inorganic acid-induced low pH in *S*. *cerevisiae* (Cabib et al., 1989). Nevertheless, an increase of cell wall polysaccharide content was also reported, in particular an increase in chitin and β1,6-glucans under heat stress and an increase in the β-glucans content under acetic acid stress in *S*. *cerevisiae* (Schiavone et al., 2014; Ribeiro et al., 2021). It is noteworthy to take in consideration that the methods used for cell wall polysaccharides quantification were not the same in different articles and both the levels of stress and the adaptation phase of the cells examined (early response, cells adapted to the stress) could be different or not clearly reported.

## Improvement of yeast tolerance to multiple stresses involving cell wall engineering

A few successful examples of the alteration of the physicochemical properties of the cell wall either by the genetic engineering of the yeast cell or by the adaptive laboratory evolution (ALE) of yeast cells leading to the increase of tolerance to stress(es) have been reported in the literature.

As referred above, through chemogenomic analyses, it was demonstrated that the expression of genes related with chitin and glucan synthesis, namely *CHS1* (chitin), *FKS1* and *ACF2* (β1,3-glucans), *KRE6* (β1,6-glucans), and others involved in cell wall integrity (*PUN1*) and cell wall organization (*SIT4*), are required for maximum tolerance to ethanol in *S. cerevisiae* (Kubota et al., 2004; Auesukaree et al., 2009; Teixeira et al., 2009; Mota et al., 2021). Superior fermentation performance of lignocellulosic hydrolysates was reported for a recombinant *S. cerevisiae* WXY70 strain overexpressing the *CCW12* gene, encoding a cell wall mannoprotein, compared to the control strain, and *CCW12* expression was found to improve cell wall stability and tolerance to the growth inhibitors present (Kong et al., 2021). The deletion of *GAL6*, encoding a cysteine aminopeptidase with homocysteine-thiolactonase activity, was found to lead to improved growth and higher viability of *S. cerevisiae*, in the presence of ethanol stress (Yazawa et al., 2007). The *gal6*Δ cells showed increased resistance to zymolyase activity indicating the occurrence of structural changes in the cell wall (Yazawa et al., 2007). A marked increased tolerance to ethanol stress associated with transcription rewiring involving cell wall synthesis was also identified in the highly–ethanol tolerant strain *K. marxianus* FIM1 obtained by ALE in an ethanol supplemented medium (Mo et al., 2019). Among the identified changes at the transcriptional level were the alterations related with the upregulation of cell wall metabolism involving, in particular, chitin synthesis (*CHS3*), β1,6-glucan synthesis (*KNH1*), glucanosyltransferases activity involved in β1,3-glucan branching and elongation (*GAS4*) and the cell wall integrity pathway (*BCK1, MID2, WSC3*) (Mo et al., 2019).

Numerous genes encoding proteins required for cell wall polysaccharides synthesis (*FKS1, ROM2, KRE6, CHS1, CHS5*), cell wall remodeling (*GAS1*) and protein mannosylation (*MNN2, MNN9, MNN11, KTR4*) were found to be determinants of acetic acid stress tolerance by chemogenomic analyses (Kawahata et al., 2006; Mira et al., 2010b). The increased expression of the *HAA1* gene, encoding a transcription factor and a major determinant of acetic acid tolerance in *S. cerevisiae*, led to the improvement of cell wall robustness under acetic acid stress, as suggested by the decreased susceptibility of the cell wall to lyticase activity mediated disruption (Cunha et al., 2018). Through Haa1 amino acid sequence engineering, a single amino acid exchange at position 135 (serine to phenylalanine) was found to lead to the upregulation of genes of the Haa1-regulon, increasing acetic acid tolerance, in particular *YGP1* (Swinnen et al., 2017). The deletion of *ATG22*, encoding a vacuolar membrane protein that mediates the efflux of amino acids resulting from autophagic protein degradation, was found to delay programmed cell death (PDC) induced by acetic acid. The deletion of *ATG22* contributes to the maintenance of cell wall integrity, by preventing the decrease in total cell wall polysaccharides induced by PDC caused by severe acetic acid stress, increasing the transcript levels of CWI pathway genes (Yang et al., 2006; Hu et al., 2019). This suggested *ATG22* as a potential target for genetic engineering strategies to improve yeast cell wall robustness and tolerance to acetic acid and other industrial stresses. Enhanced tolerance to both acetic and formic acids at low pH (pH 2.4 or below) by expressing the *GAS1* gene of *Issatchenkia orientalis* in *S. cerevisiae* was reported (Wada et al., 2020). Also, the overexpression of *GAS1* in *S. cerevisiae* led to increased lactic acid productivity at low pH (Zhong et al., 2021), reinforcing the importance of *GAS1*, encoding a beta-1,3-glucanosyltransferase, in this context (Baek et al., 2017).

As previously referred, chemogenomic studies demonstrated that genes related with cell wall polysaccharide synthesis (*FKS1, KRE6*) and cell wall mannosylation (*MNN10, ANP1*) are determinants of tolerance to osmotic stress (high glucose concentrations) in *S. cerevisiae* (Ando et al., 2006; Teixeira et al., 2010). In *Pichia pastoris* (now *Komagataella phaffii*), the deletion of *YPS7*, encoding a putative GPI-linked aspartyl protease, led to increased osmotic tolerance and this gene was proposed as a promising molecular target for the engineering of yeast robustness (Guan et al., 2012). This species is used to produce heterologous proteins in the pharmaceutical and food industry.

In a laboratory adaptively evolved *K. marxianus* strain exhibiting a significantly improved tolerance to high temperatures, a single nucleotide polymorphism was found in the coding region of the exoglucanase gene *EXGI*, required for cell wall remodeling (Ai et al., 2022).

## Concluding remarks

The important role played by the cell wall in the adaptation and tolerance of yeasts to different stresses of biotechnological interest emerges from this review article. Although several evidences support this idea, the truth is that these evidences largely come from genome-wide analyses of the response of yeasts to various stresses. Furthermore, the experimental conditions and levels of stress used are in general different and only part of the studies involves a time-course analysis covering the various stages of adaptation and growth under stress. Therefore, it is likely that the apparent divergences reported are due to this fact.

This review article also provides information on how the different signaling pathways coordinate to elicit changes at the level of the yeast cell wall in response to different relevant stresses. However, it is important to highlight that, in industrial bioprocesses, several stresses are often present simultaneously, which further complicates the goal of providing a comprehensive description of what happens during adaptation to adverse process conditions. It is noteworthy to take in consideration that yeast cells exposed to mild stress develop tolerance not only to higher doses of the same stress, but also to stress caused by other agents. This phenomenon, known as cross-protection, is based on the existence of integrating mechanisms that senses and responds to different forms of stress (Estruch, 2000). Concerning the cell wall, the physiological observations reported and the integrated molecular responses here described provide the basis for the involvement of this dynamic organelle in cross-stress protection.

Most of the review paper is dedicated to the model yeast and cell factory *Saccharomyces cerevisiae*. However, the scarce available information in the literature concerning non-conventional yeast species of biotechnological relevance is also mentioned throughout the text. A deeper understanding of the nature of the molecular response and the changes occurring at the cell envelope level would be valuable and allow the development of more rational strategies to construct superior yeasts for biotechnology and to control the activity of food spoiling yeasts. Genome-wide analyses are contributing to identify a wealth of cell wall-related promising targets for the improvement of yeast tolerance. However, more in depth molecular and cellular studies are instrumental to better understand the somewhat overlooked role of the cell wall in tolerance to multiple stresses in yeasts.

## Author contributions

IS-C, RR, and NB-M conceived and designed the study. RR and NB-M performed the literature search, prepared the figures, and wrote the first manuscript draft. IS-C and RR completed and revised the manuscript drafts. All authors approved the final manuscript.
